# Size‐dependent and environment‐mediated shifts in leaf traits of a deciduous tree species in a subtropical forest

**DOI:** 10.1002/ece3.8516

**Published:** 2022-01-12

**Authors:** Jie Zheng, Ya Jiang, Hong Qian, Yanjiao Mao, Chao Zhang, Xiaoxin Tang, Yi Jin, Yin Yi

**Affiliations:** ^1^ Key Laboratory of National Forestry and Grassland Administration on Biodiversity Conservation in Karst Mountainous Areas of Southwestern China Guizhou Normal University Guiyang China; ^2^ School of Life Sciences Guizhou Normal University Guiyang China; ^3^ Research and Collections Center Illinois State Museum Springfield Illinois USA; ^4^ Key Laboratory of Plant Physiology and Developmental Regulation of Guizhou Province Guizhou Normal University Guiyang China

**Keywords:** *Clausena dunniana*, intraspecific trait variation, karst forest, leaf area, Maolan National Nature Reserve, specific leaf area

## Abstract

**Aims:**

Understanding the joint effects of plant development and environment on shifts of intraspecific leaf traits will advance the understandings of the causes of intraspecific trait variation. We address this question by focusing on a widespread species *Clausena dunniana* in a subtropical broad‐leaved forest.

**Methods:**

We sampled 262 individuals of *C*. *dunniana* at two major topographic habitat types, the slope and hilltop, within the karst forests in Maolan Nature Reserve in southwestern China. We measured individual plant level leaf traits (i.e., specific leaf area (SLA), leaf area, leaf dry‐matter content (LDMC), and leaf thickness) that are associated with plant resource‐use strategies. We adopted a linear mixed‐effects model in which the plant size (i.e., the first principal component of plant basal diameter and plant height) and environmental factors (i.e., topographic habitat, canopy height, and rock‐bareness) were used as independent variables, to estimate their influences on the shifts of leaf traits.

**Key Results:**

We found that (1) plant size and the environmental factors independently drove the intraspecific leaf trait shifts of *C*. *dunniana*, of which plant size explained less variances than environmental factors. (2) With increasing plant size, *C*. *dunniana* individuals had increasingly smaller SLA but larger sized leaves. (3) The most influential environmental factor was topographic habitat; it drove the shifts of all the four traits examined. *Clausena dunniana* individuals on hilltops had leaf traits representing more conservative resource‐use strategies (e.g., smaller SLA, higher LDMC) than individuals on slopes. On top of that, local‐scale environmental factors further modified leaf trait shifts.

**Conclusions:**

Plant size and environment independently shaped the variations in intraspecific leaf traits of *C*. *dunniana* in the subtropical karst forest of Maolan. Compared with plant size, the environment played a more critical role in shaping intraspecific leaf trait variations, and potentially also the underlying individual‐level plant resource‐use strategies.

## Graphical abstract

Plant size and environment independently shaped the variations in intraspecific leaf traits of *Clausena dunniana* in the subtropical karst forest of Maolan. Compared with plant size, the environment played a more critical role in shaping intraspecific leaf trait variations, and potentially also the underlying individual‐level plant resource‐use strategies.

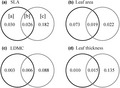

## 1 INTRODUCTION

Intraspecific trait variation is an increasingly important topic in trait‐based ecology (Bolnick et al., 2011; Moran et al., 2016; Westerband et al., 2021). Understanding how plant development (hereafter simply development) and environment jointly shape plant leaf traits, will advance the understandings of the causes of intraspecific trait variation (Shipley et al., 2016; Violle et al., 2012). In turn, it would shed light on the understandings of species distribution (Grime, 1965), the assembly of ecological communities (Jung et al., 2010; Siefert et al., 2015), and the modeling of ecological processes (Funk et al., 2017; Moran et al., 2016).

For long‐lived woody angiosperms, plant size is commonly used as a proxy of developmental stage (e.g., He & Yan, 2018; Martin & Thomas, 2013). In the forest, as a plant increases in size, it generally faces increasingly higher irradiance and hydraulic resistance at higher forest strata (Rozendaal et al., 2006; Thomas & Bazzaz, 1999), as a result, leaf traits usually show corresponding shifts toward more conservative resource‐use strategy (e.g., Cavaleri et al., 2010; Dayrell et al., 2018; He & Yan, 2018; Ishida et al., 2005). Larger conspecific individuals usually have smaller sized leaves (Koch et al., 2004), smaller specific leaf area (SLA) (Kenzo et al., 2015; Thomas & Winner, 2002), as well as higher leaf dry‐matter content (LDMC) (Park et al., 2019). These size‐dependent trait shifts are interpreted as plant adopting an increasingly more conservative light and water‐use strategies in response to high light and drought stress, through acclimation and plasticity. However, there are exceptional cases (e.g., He & Yan, 2018; Thomas & Ickes, 1995). For example, Thomas and Ickes (1995) found larger conspecific individuals had larger sized leaves for understory treelet species, suggesting a more liberal resource‐use strategy.

Generally, the leaf traits that represent plant resource‐use strategies are tightly linked with environmental conditions (Reich, 2014). For example, increasingly smaller SLA is likely to be found in higher light, lower water, and soil nutrient environments (Kikuzawa & Lechowicz, 2011; Poorter et al., 2009). If intraspecific leaf trait shifts on the environmental gradients follow the trends of interspecific trait shifts (e.g., Cornwell & Ackerly, 2009; Geekiyanage et al., 2018; Wright et al., 2017), one would expect to find intraspecific leaf traits showing corresponding shifts on the environmental gradients (e.g., Fajardo & Piper, 2011; Fajardo & Siefert, 2018). Intraspecific leaf traits often shift toward increasingly conservative resource‐use strategies with decreasing environmental wetness (Schmitt et al., 2020; Souza et al., 2018), decreasing soil fertility (Knops & Reinhart, 2000; Pakeman, 2013; Poorter et al., 2009), or increasing light availability (Gratani et al., 2006; Martin et al., 2020). However, some previous studies on testing intraspecific trait shifts along the environmental gradients showed mixed results (e.g., Kühn et al., 2021; Royer et al., 2008). For example, in the Canary archipelago, Kühn et al. (2021) found the shifts in intraspecific leaf traits of native and non‐native species showed contrasting patterns along an elevational gradient.

Evidently, development and environment are two major drivers of intraspecific trait variations. However, it remains unclear how development and environment jointly shape the intraspecific leaf trait shifts (Tredennick et al., 2018). On the one hand, researchers suggest that development and environment interact in mediating intraspecific trait variations, attributing to ontogenetic plasticity and acclimation to environmental heterogeneity (Russo & Kitajima, 2016). On the other hand, empirical evidence was equivocal, some found development and environment interactively (e.g., Dayrell et al., 2018), others found independently (e.g., Fortunel et al., 2020; Liu et al., 2020), in shaping the intraspecific trait variations. Furthermore, previous studies that estimated the relative importance of development and environment in shaping variations in intraspecific traits also found inconsistent results (e.g., Fajardo & Piper, 2011; Fortunel et al., 2020; Liu et al., 2020). For example, Fajardo and Piper (2011) found environment dominates over plant size in shaping leaf mass per area (LMA) of a deciduous angiosperm tree in southern Andes, but Liu et al. (2020) found that the opposite was true for a conifer tree in the forest of northern China.

An extensive zone of karst forests lies in southwestern China (Geekiyanage et al., 2018), but knowledge of karst forests leaf traits is limited (Geekiyanage et al., 2019). Under a subtropical monsoon climate, rugged topography of the karst forest results in fast soil erosion and leaching, accompany with slow soil formation on the carbonate bedrock (Li et al., 2008), together create sharp gradients of light, water, and soil nutrients (Bonacci et al., 2009; Geekiyanage et al., 2018; Guo et al., 2017). A large piece of the karst forest is preserved in Maolan National Nature Reserve (hereafter Maolan; Zhou, 1987). The karst forest of Maolan grows mainly on a geomorphological feature termed as karst peak‐cluster, which represents a cluster of several steep‐sided karst hills sharing a common base (Sweeting, 1995). The peak‐cluster karst forest of Maolan can be classified into a series of topographic habitats, starting from the high light, low moisture, and infertile hilltop toward low light, moist, and fertile slope and foothill (Gan & Mu, 1987; Zhang & Zhang, 1987; Zhou, 1987). Apart from this regular pattern, smaller local‐scale environmental conditions in the karst forests are largely irregular, such as canopy condition (e.g., canopy height; Long et al., 2005) and microtopography (e.g., rock outcrops; Zhang et al., 2010). The karst forest experiences high frequency of canopy disturbance (i.e., treefall gap; Long et al., 2005), resulting in varied canopy height and light penetration to forest lower strata. Rocky outcrops (or rock‐bareness) are a common feature and constrain the availability of soil water and nutrients (Huang et al., 2009; Zhang et al., 2007). Previous studies have confirmed the effects of these environmental factors on plant distribution and specialization (e.g., Geekiyanage et al., 2018; Guo et al., 2017; Li et al., 2019; Long et al., 2005; Zhang et al., 2013). However, it remains unclear how these environmental factors and plant development jointly influence the variations in intraspecific traits in the karst forests.

Here, we explore how development and environment jointly (including their interactions and relative importance) drive the shifts of intraspecific leaf traits of *Clausena dunniana*, which is a common species widely distributed in the understory of the subtropical broad‐leaved forest on the karst hills of Maolan. Specifically, we test the joint effects of plant size, topographic habitat, and local environmental conditions on the shifts of leaf traits (i.e., SLA, leaf area (LA), LDMC, leaf thickness (*L*_th_)) of *C*. *dunniana*. We predict that leaf traits of *C*. *dunniana* would shift toward more conservative resource‐use strategies (i.e., smaller leaf area and SLA, higher LDMC and leaf thickness) with increasing plant size, and this trend be more pronounced in high light, low moisture, and infertile environments (i.e., hilltop, low canopy, and high rock‐bareness locations), compared to dark, moist, and fertile environments (e.g., slope, high canopy, and low rock‐bareness locations).

## 2 MATERIALS AND METHODS

### 2.1 Study site and species

The Maolan National Nature Reserve (25°09′20″–25°20′50″N, 107°52′10″–108°05′40″E) is located in Libo County of Guizhou Province in southwestern China. Local annual mean temperature is 15.3°C, with January mean of 5.2°C and July mean of 23.5°C. Mean annual precipitation is 1,752.5 mm. The bedrock of the reserve consists of Carboniferous and Permian limestones and dolomites, with elevation varying from 430 to 1,078 m a.s.l. (Zhou, 1987). In the karst peak‐cluster of Maolan, the height from the hilltop (i.e., the top of individual hill that forms the peak‐cluster) to the bottom ranges from 150 to 300 m (Li & Li, 1987). More than 90% of the reserve is forested (Zhou, 1987). The study species *Clausena dunniana* (Rutaceae) is a deciduous compound leaved plant, with an adult height of 2–5 m. Its compound leaf is composed of 5–15 leaflets. The leaflet blade is ovate to lanceolate in shape, with a length of 4–10 cm and a width of 2–5 cm. *Clausena dunniana* is mainly distributed at 300–1,500 m a.s.l. in montane forests in southwestern China (Flora of China Editorial Committee, 2004). In the forests of Maolan, *C*. *dunniana* is a dominant tree species distributed on the whole topographic gradients of the peak‐cluster, with more even distribution across topographic habitats from hilltop to bottom, compared with most other tree species (Qin et al., 2018).

### 2.2 Plant sampling and trait measurement

**FIGURE 1:**
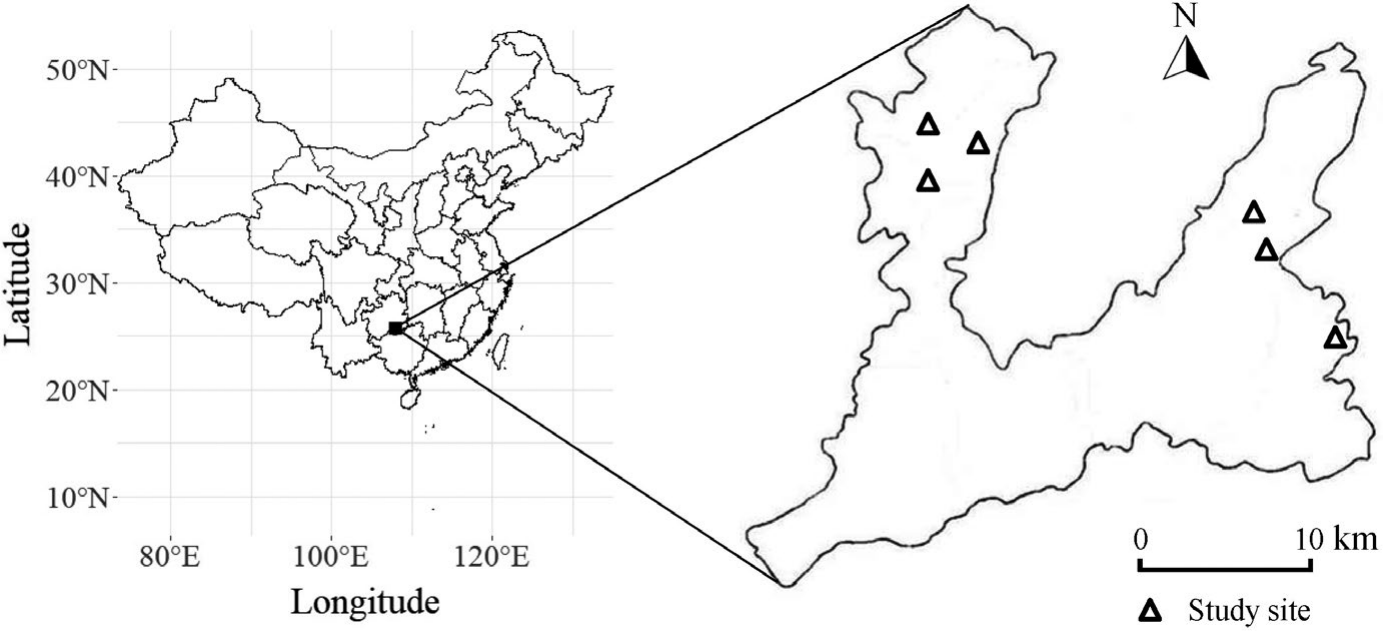
Location of the study sites in Maolan National Nature Reserve in China

In late July of 2020, using a strip transect (60 m × 10 m) method (Buckland et al., 2007), we sampled 262 individuals of *C*. *dunniana* at two major topographic habitat types, the slope (152 individuals) and the hilltop (110 individuals), within the forests of six peak‐clusters (as sites, sample size ranged from 33 to 66 individuals) evenly distributed in two regions (sample size were 144 and 118 individuals respectively) about 10 km apart within the reserve (Figure 1). The slopes are located between the hilltop and the foothill. The hilltops include the top of the hills as well as ridges that connect nearby hilltops. These hilltops had thinner soil, higher soil calcium, and lower soil phosphorous concentrations compared with the slopes (Jin et al. unpublished data). For each sampled individual plant, we estimated its neighborhood canopy height and rock‐bareness rate, and used it to represent local‐scale environmental conditions. We estimated canopy height using a measuring pole mounted with a rod level. Specifically, we estimated the height of the highest living foliage directly above the focal plant, and over four points at five‐meter radius from the focal plant at four random directions, the average of the five measurements was used to represent the neighborhood canopy height (Welden et al., 1991). Rock‐bareness rate represents the percentage of ground surface covered with rocky outcrops within five meters of the focal plant, and was visually estimated to 10 levels (Zhang et al., 2013): 1 (<10%), 2 (10%–20%), 3 (20%–30%), 4 (30%–40%), 5 (40%–50%), 6 (50%–60%), 7 (60%–70%), 8 (70%–80%), 9 (80%–90%), and 10 (90%–100%).

For each sampled individual plant, we measured the basal diameter (0.1 m aboveground) of its stem/trunk using an electronic digital vernier caliper (PD‐151, Pro'sKit, Shanghai, China) for individuals with basal diameter <5 cm, or using a diameter tape for larger individuals (i.e., basal diameter ≥5 cm). We measured the height of the sampled individual using a pole with a rod level. With respect to leaf sampling, we randomly sampled 2–10 fully expanded intact compound leaves located at the outer layer of the upper portion of the crown of the plant using a tree pruner (LZ5625, VMP, Shandong, China). The sampled leaves were placed in black plastic bags, stored in portable refrigerating box at above 0°C temperature and transported back to the laboratory in the same day. Then, the leaves were placed in fresh water for rehydration overnight. In the next day, the sampled compound leaves were scanned using a flatbed scanner, and leaf area was measured as the sum of one‐sided projected fresh lamina surface area of all the leaflets without petiole and rachis, using ImageJ software (Schneider et al., 2012). For each sampled compound leaf, three leaflets were randomly selected. For each leaflet, thickness was measured as the mean of three measurements taken between the tip and base of the lamina, avoiding the major veins, using an electronic digital vernier caliper (PD‐151, Pro'sKit, Shanghai, China). Then the average thickness of the three leaflets was calculated to represent the leaf thickness of the compound leaf. The average of leaf area and leaf thickness of the sampled compound leaves were used to represent the leaf area and leaf thickness of the sampled individual plant, respectively. After that, the fresh weight of all the leaflets of the sampled compound leaves with petiole and rachis being removed was measured in an electronic scale (BP‐223A+, SETPRO, Shanghai, China) for each individual plant. The leaflets were then dried in an oven (101‐3BS, LICHEN, Shanghai, China) at 70°C for 72 h, and the dry leaf mass was measured in an electronic scale (BP‐223A+, SETPRO, Shanghai, China). Specific leaf area was calculated as the sum of leaf area divided by the sum of dry leaf mass of the sampled compound leaves for each individual plant. Leaf dry‐matter content was calculated as the sum of dry leaf mass divided by the sum of fresh weight of the sampled compound leaves for each individual plant.

### 2.3 Statistical analyses

The plant size‐dependent and environmental factors (i.e., topographic habitat, canopy height, and rock‐bareness rate) mediated shifts in leaf traits (i.e., SLA, leaf area, LDMC, and leaf thickness) were estimated by linear mixed‐effects model using the “lmer” function in the *lmerTest* package in R 3.6.1 (R Core Team, 2019). For the two factors used to represent plant size, basal diameter was highly correlated with plant height (Pearson's *r* = .803, *p* < .001). A principal component analysis (PCA) was conducted on basal diameter and plant height, and the first principal component of the PCA, which explained 98.4% of the total variance, was used to represent plant size, larger value of the first principal component represents larger sized plant. Correlations between the predictors (i.e., plant size, topographic habitat, canopy height, rock‐bareness rate) were estimated, and no high correlation (Pearson' *r* ≥ .5 or ≤ −.5) was detected.

The response (dependent) variable of a linear mixed‐effects model was the SLA, LA, LDMC, or *L*_th_ (*t_ijk_*) of individual plant *i* at site *j* of region *k*. Variation in *t_ijk_* was modeled as a function of plant size (*PS*), topographic habitat (*TH*), and local environmental conditions (i.e., canopy height (*CH*), and rock‐bareness rate (*RB*)), plus the two‐way interactions between *PS* and the three environmental factors, as fixed effects. The two‐way interactions were included to examine if size‐dependent leaf trait shifts were modified by the environment. A random intercept (*μ_jk_*) was specified for site *j* nested within region *k*, as random effect. The model structure was:

$$t_{\mathrm{ijk}}=\alpha +\beta_{1}{\text{PS}}_{\mathrm{ijk}}+\beta_{2}{\text{TH}}_{\mathrm{jk}}+\beta_{3}{\text{CH}}_{\mathrm{ijk}}+\beta_{4}{\text{RB}}_{\mathrm{ijk}}+\gamma_{1}{\text{PS}}_{\mathrm{ijk}}{\text{TH}}_{\mathrm{jk}}+\gamma_{2}{\text{PS}}_{\mathrm{ijk}}{\text{CH}}_{\mathrm{ijk}}+\gamma_{3}{\text{PS}}_{\mathrm{ijk}}{\text{RB}}_{\mathrm{ijk}}+\mu_{\mathrm{jk}}$$

where *β*_1_ through *β*_4_ are fixed‐effect coefficients representing variation in SLA, LA, LDMC, or *L*_th_ due to variation in plant size, topographic habitat, canopy height, and rock‐bareness rate, respectively; *γ* represents fixed‐effect coefficient for second‐order interaction of these factors; and *α* represents the mean trait value of the individual plant averaged over all sites and regions. The random term *μ_jk_* was assumed to be normally distributed as $\mu_{\mathrm{jk}}∼N\left(0,\sigma_{\mu }\right)$. Before analysis, all continuous predictor variables were standardized by subtracting the mean and dividing by the standard deviation (i.e., mean = 0, SD = 1; Table 1). We tested variance inflation factors (VIF) for the predictor variables as well as their two‐way interactions for the four models using the “vif.mer” function in the *ieco* package (Helmus, 2021) in R; we found all the VIF values were less than 3 for all the four models, which were substantially smaller than 10 (Dormann et al., 2013), suggesting that multicollinearity among the explanatory variables is not an issue in our study.

**TABLE 1:** Summary of the predictor and response variables in the fixed‐effects portion of the linear mixed‐effects models

Predictor variables	
Discrete	Levels
Topographic habitat	Slope, hilltop
Rock‐bareness rate	1, 2, 3, 4, 5, 6, 7, 8, 9, 10

Continuous	Range	Mean	SD
Canopy height (m)	2.40–19.50	9.17	4.90
Plant size	−1.07–3.04	0	1.00

Response variables			
Continuous	Range	Mean	SD
SLA (cm^2^ g^−1^)	62.67–300.20	125.68	34.76
Leaf area (cm^2^)	27.36–232.46	83.67	35.47
LDMC (%)	21.6–69.1	38.29	5.60
Leaf thickness (mm)	0.09–0.38	0.26	0.05

Abbreviations: LDMC, leaf dry‐matter content; SLA, specific leaf area.

The model selection was performed using a backward elimination approach, starting with the full model (Jin et al., 2018). The fixed terms that produced the largest drop in Akaike information criterion corrected for small sample size (AICc) value were sequentially deleted, and the model with the lowest AICc was considered the most supported model. AIC estimation based on maximum likelihood (ML) was adopted for fixed‐effect model comparisons. The procedure was conducted with the “model.sel” function in the *MuMIn* package (Bartoń, 2019). After model selection was done, coefficients of the most supported model were estimated using restricted maximum likelihood (REML). Model assumptions of normality of model residuals were met in all models. Model fit was estimated for the most supported model using marginal ($R_{m}^{2}$) and conditional ($R_{c}^{2}$) pseudo‐*R* ^2^ values (Nakagawa & Schielzeth, 2013) computed with the “r.squaredGLMM” function in the *MuMIn* package (Bartoń, 2019). Furthermore, a variation partitioning analysis was conducted to estimate the relative importance of plant size and the three environmental factors in explaining the variances in the leaf traits, using the “varpart” function in the *vegan* package. All the analyses were conducted in R 3.6.1 (R Core Team, 2019).

## 3 RESULTS

**FIGURE 2:**
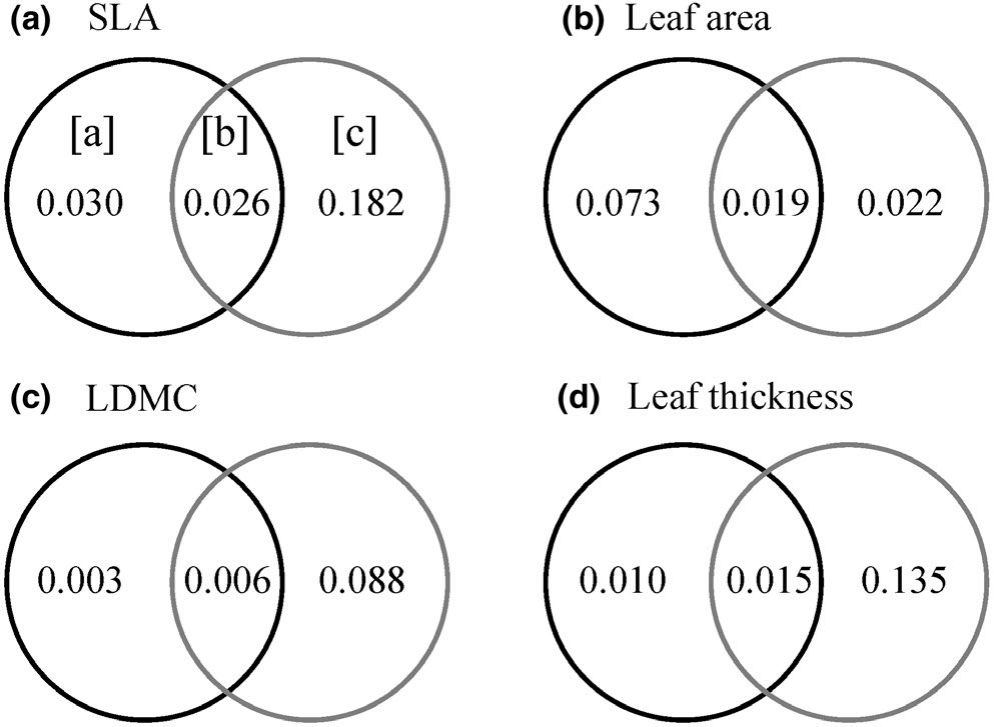
Venn diagram of proportion of variance of the leaf traits explained by plant size and the environmental factors SLA, specific leaf area; LDMC, leaf dry‐matter content. Portion (a) is explained only by plant size, portion (b) is explained by both plant size and the environment, (c) is explained only by the environment

The most supported models of the shifts of the four leaf traits explained significant amounts of variance in the data. The fixed part of the models explained a proportion of the variance ($R_{m}^{2}$) ranged from 0.054 to 0.211, the total proportion of the variance explained by the models ($R_{c}^{2}$) including the random part ranged from 0.146 to 0.491 (Table 2). However, the most supported models retained no two‐way interactions, indicating that there was lack of significant interaction effects between plant size and environment on the leaf trait shifts of *C*. *dunniana* (Table 2). In the most supported model, the shifts of SLA and leaf area were driven by both plant size and environmental factors, the shifts of LDMC and leaf thickness were driven only by environmental factors (Table 2). Furthermore, variation partitioning analysis showed that plant size explained a smaller proportion of the variance in three of the four leaf traits than did environmental factors (Figure 2).

**TABLE 2:** Coefficients of plant size and environmental factors on the shifts in individual‐level leaf traits as estimated by the most supported linear mixed‐effects model

Fixed terms	SLA	Leaf area	LDMC	Leaf thickness
Plant size	−5.76**	9.29***		
Topographic habitat (hilltop)	−16.77***	9.11*	2.64***	0.03***
Canopy height	4.31			−0.01**
Rock‐bareness rate	−5.58*			
Plant size × Topographic habitat (hilltop)				
Plant size × Canopy height				
Plant size × Rock‐bareness rate				

Note

Effects of factors not retained in the most supported model are not shown. Pseudo marginal *R* ^2^ ($R_{m}^{2}$) for the most supported models of SLA, leaf area, LDMC, and leaf thickness was 0.212, 0.088, 0.053, and 0.159, respectively; pseudo conditional *R* ^2^ ($R_{c}^{2}$) for the most supported models of SLA, leaf area, LDMC, and leaf thickness was 0.271, 0.159, 0.238, and 0.441, respectively. Significance levels: *p < .05; **p < .01; ***p < .001.

Abbreviations: LDMC, leaf dry‐matter content; SLA, specific leaf area.

**FIGURE 3:**
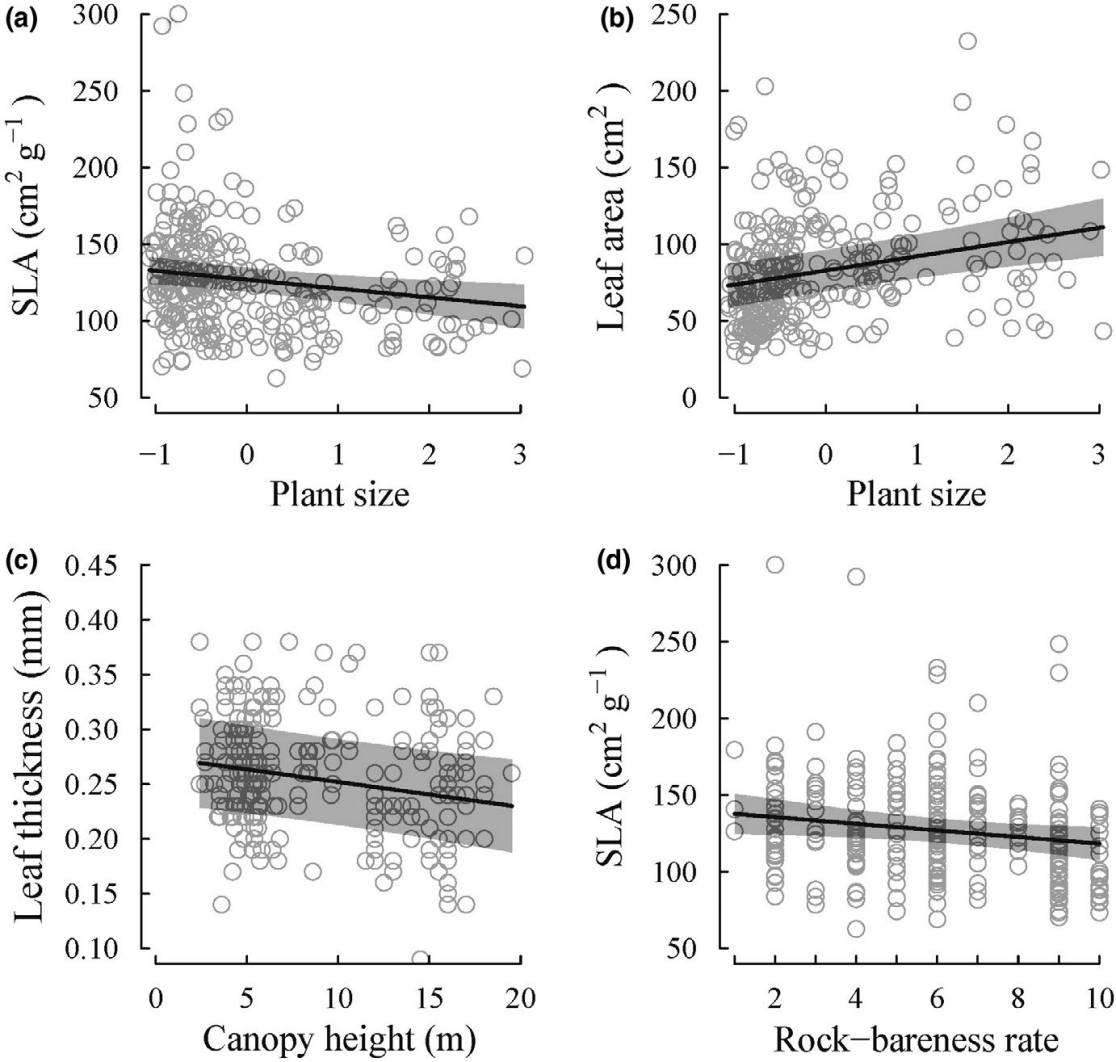
Shifts in leaf traits in response to plant size, canopy height, and rock‐bareness rate as estimated by the most supported linear mixed‐effects models SLA, specific leaf area; LDMC, leaf dry‐matter content. Each empty circle represents an individual plant. The fitted line represents predicted values by the most supported linear mixed‐effects model as shown in Table 2. The shaded region indicates 95% confidence interval of the predicted values

**FIGURE 4:**
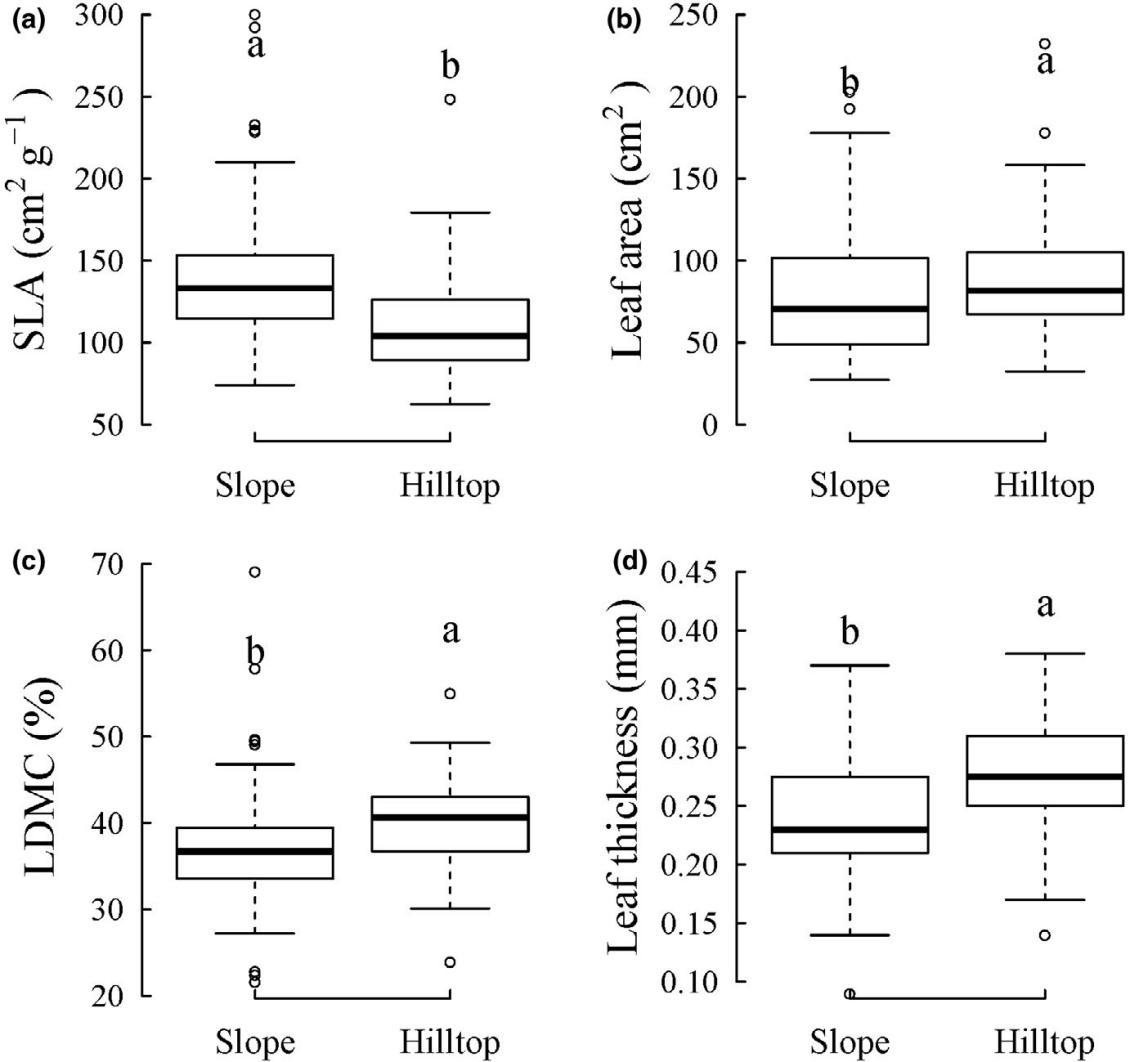
Differences in leaf traits between topographic habitats as estimated by the most supported linear mixed‐effects models SLA, specific leaf area; LDMC, leaf dry‐matter content. Boxplot shows the median, the first and third quartiles of the observed leaf traits, with whiskers extending to 1.5 times the interquartile range, and empty circles represent outliers. Significant difference (*p* < .05 as estimated by the most supported linear mixed‐effects models in Table 2) between groups are indicated by different lowercase letters above the boxplots

With respect to plant size effects on leaf trait shifts, we found that larger sized *C*. *dunniana* individuals have smaller SLA and larger sized leaves (Figure 3a,b and Table 2). The plant size was not retained in the most supported model of LDMC and leaf thickness, suggesting a lack of plant size‐related shifts of these two leaf traits (Table 2). On the other hand, among the three environmental factors, the most important factor was topographic habitat, it propelled the shifts of all the four leaf traits we examined (Table 2). Specifically, comparing between topographic habitats, *C*. *dunniana* individuals in the slope habitat had larger SLA, lower LDMC, smaller sized, and thinner leaves than *C*. *dunniana* individuals on hilltops (Figure 4 and Table 2). On top of topographic habitat influence, leaf traits were further modified by local‐scale environmental conditions. Canopy height was retained in the most supported model of leaf thickness, and rock‐bareness rate was retained in the most supported model of SLA (Table 2). Specifically, at locations with increasingly higher canopies, *C*. *dunniana* individuals had increasingly thinner leaves (Figure 3c and Table 2); at locations with increasingly higher rock‐bareness rates, *C*. *dunniana* individuals had increasingly smaller SLA (Figure 3d and Table 2).

## 4 DISCUSSION

In this study, we explored the joint effects of plant development and environment on intraspecific leaf trait variations for a tree species in a subtropical broad‐leaved forest in the Maolan Nature Reserve in southwestern China. We found that plant size and environmental factors independently influenced the intraspecific leaf trait variations in *C*. *dunniana*. Our finding is in line with the findings of Liu et al. (2020) and Fortunel et al. (2020). We suspect the lack of interactive effects of plant size and environmental factors, was possibly due to an early established leaf trait divergence shortly after seedling emergence in response to different environmental conditions (Larson et al., 2020; Reader et al., 1993), or due to inherent developmental constraints driving differential ontogenetic trajectories (Fortunel et al., 2020).

With respect to the independent effects of plant size and environment on the four leaf traits of *C*. *dunniana* examined, we found that size‐dependent shifts were less frequent and overall, less influential than environment‐mediated shifts. Our finding is consistent with the finding of environment dominated over plant size in controlling leaf trait of a widespread tree species *Nothofagus pumilio* in the southern Andes of Chile (Fajardo & Piper, 2011), but our finding is contrary to a previous study on a conifer tree species *Pinus koraiensis* in a temperate forest in northern China (Liu et al., 2020). Since the relative importance of environment in driving intraspecific trait variations likely increase with the spatial extent and environment heterogeneity of a study system (Spasojevic et al., 2016), we suspect that the inconsistent results of these three studies at least partly stemmed from the difference in spatial extent and environmental heterogeneity encompassed. Specifically, Liu et al.'s study was conducted in a 9‐ha forest plot lying on a gentle topography (Xu & Jin, 2012), which might have led to the relatively small role of environment in driving intraspecific leaf trait shifts. On the other hand, Fajardo and Piper's study covered large elevation ranges located at two distant regions, and the present study was conducted on rugged karst peak‐clusters. These two studies covered much larger spatial extents and probably also larger environmental heterogeneities, hence facilitated more prominent roles of environment in driving intraspecific leaf trait shifts.

Generally, forest plants face increasingly higher light and hydraulic stresses as plant sizes increase and are closer to the canopy (Rozendaal et al., 2006; Thomas & Bazzaz, 1999). During the process of development, these stresses are expected to push the leaf traits toward greater conservative resource‐use strategies (Dayrell et al., 2018). Our finding of the pattern of SLA declining with increasing plant size of *C*. *dunniana*, agrees with this expectation and is in line with the pattern widely reported from forests in other parts of the world (e.g., Kenzo et al., 2015; Martin & Thomas, 2013; Park et al., 2019; Thomas & Winner, 2002). However, the leaf area of *C*. *dunniana* enlarged with increasing plant size, which is contrary to expectation (Koch et al., 2004). As a matter of fact, the size‐dependent increase in leaf area is not uncommon in forest plants (e.g., He & Yan, 2018; Ishida et al., 2005; Thomas & Ickes, 1995). For example, Thomas and Ickes (1995) found a differential size‐dependent shift in leaf area between canopy and understory plant species in a Malaysian rain forest. Specifically, the leaf area of understory treelet species tended to increase with increasing plant size, whereas canopy tree species tended to show the reverse pattern in their study. Thomas and Ickes (1995) suggested that the contrary patterns might be partly due to the difference between canopy and understory species in adult stature and crown exposure. Canopy species are expected to have high crown exposure as adults, and they invest heavily on vertical growth and show leaf traits that are increasingly advantageous in higher irradiance environments (e.g., smaller sized leaves) toward the canopy. On the other hand, the small asymptotic heights of understory species mean low opportunity of high crown exposure as adults, and they invest increasingly more to horizontal growth, such as adopting leaf traits that capture more irradiance in understory environment (e.g., larger sized leaves).

Another possible driver of size‐dependent shifts in leaf traits is reproductive onset (Thomas, 2010). Specifically, before reproductive onset, a plant invests resources mainly on vegetative growth, and adopts an increasingly acquisitive resource‐use strategy as the plant meets with increasing light availability and achieving higher water‐use efficiency during its development. After reproductive onset, the plant allocates more resource to reproduction, and switches to a more conservative leaf resource‐use strategy. Under this scenario, one is expected to find a unimodal pattern of leaf trait shifts during development, peaked approximately at the reproductive onset stage (Thomas, 2010). In the present study, our sampling covered a wide plant size range (with a basal diameter range of 0.36–15.7 cm) that probably extends beyond the reproductive onset stage of *C*. *dunniana*, yet the monotonic shifts of leaf traits were apparent, therefore, we suspect that reproduction might not be an influential factor of plant size‐related leaf trait shifts for *C*. *dunniana*.

Previous studies conducted in the karst forests found topography was closely related to plant distribution (Guo et al., 2017; Zhang et al., 2013) and trait specialization (Geekiyanage et al., 2018). In this study, we found topography was critical for leaf trait shifts of *C*. *dunniana*, which extended our understanding of the topographic effects on leaf traits in the karst forests from the interspecific level (Geekiyanage et al., 2018) to the intraspecific level. Specifically, Geekiyanage et al. (2018) found that the plant species specialized in karst hilltops had leaf traits representing more conservative resource‐use strategy compared with species specialized in slopes and foothills. We found this topography‐related leaf trait pattern also holds for the conspecifics, shown as the individuals of *C*. *dunniana* on hilltops had leaf trait values representing more conservative resource‐use strategy, such as lower SLA and higher LDMC, compared to individuals on slopes.

Local‐scale rock‐bareness rate mediated effects on leaf trait shifts likely stemmed from underlying water and edaphic conditions (Zhang et al., 2007). Since rock‐bareness constrains the availability of water and soil nutrients in the karst forest (Huang et al., 2009), the decline of SLA with increasing rock‐bareness in the neighborhood was possibly a plant response to greater water and edaphic stresses, and shifted the ecological strategies toward more conservative resource‐use strategies. Furthermore, as the optimization theory prediction of leaf thickness be positively related to light (Pérez‐Harguindeguy et al., 2016), the decrease in leaf thickness with increasing local‐scale canopy height, as was found in this study, is possibly due to a response to the reduction in exposure and light with increasing canopy height (Clark et al., 1996).

In conclusion, our study shows that plant size and environment independently shaped the intraspecific leaf trait variations in *C*. *dunniana* in the subtropical karst forest of Maolan. Compared with plant size, the environment of the karst forest played a more critical role in shaping the variations in intraspecific leaf traits, and potentially also in shaping the underlying individual‐level plant resource‐use strategies. Deeper understandings of the ecological significance of these variations in intraspecific leaf traits, such as their contribution to individual performance as well as population dynamics of *C*. *dunniana*, requires further investigation.

## CONFLICT OF INTEREST

All authors declare no conflicts of interest.

## AUTHOR CONTRIBUTIONS

**Jie Zheng:** Data curation (equal); formal analysis (equal); investigation (equal); methodology (equal); writing – original draft (equal). **Ya Jiang:** Data curation (equal); formal analysis (equal); investigation (equal); methodology (equal); writing – original draft (equal). **Hong Qian:** Writing – original draft (supporting); writing – review and editing (supporting). **Yanjiao Mao:** Data curation (supporting); investigation (supporting); writing – original draft (supporting). **Chao Zhang:** Investigation (supporting); writing – review and editing (supporting). **Xiao‐Xin Tang:** Funding acquisition (supporting); writing – review and editing (supporting). **Yi Jin:** Conceptualization (lead); formal analysis (supporting); funding acquisition (lead); investigation (supporting); methodology (supporting); project administration (supporting); writing – original draft (lead). **Yin Yi:** Funding acquisition (supporting); writing – review and editing (supporting).

## Data Availability

The data used in this study are archived in Dryad Digital Repository: https://doi.org/10.5061/dryad.rfj6q57c4.
